# Supplementary material for: Blunt-End Driven Re-entrant Ordering in Quasi Two-Dimensional Dispersions of Spherical DNA Brushes
Source: ACS Nano. 2022 Feb 7;16(2):2133–46. doi: 10.1021/acsnano.1c07799 (PMC8867906; doi:10.1021/acsnano.1c07799)
Supplement: Supplementary file 1 — nn1c07799_si_001.pdf [file nn1c07799_si_001.pdf]

Supporting Information for

Blunt-end driven re-entrant ordering in quasi two-dimensional dispersions of spherical DNA brushes

Ivany Romero-Sanchez,[1,2]† Ilian Pihlajamaa,[3,4]† Natasa Adžić,[3] Laura E. Castellano,[2] Emmanuel Stiakakis,[5] Christos N. Likos,[3] Marco Laurati[1]**

[1]Dipartimento di Chimica & CSGI, Università di Firenze, 50019 Sesto Fiorentino, Italy

[2]División de Ciencias e Ingenierías, Universidad de Guanajuato, 37150 León, Mexico

[3]Faculty of Physics, University of Vienna, Bolzmanngasse 5, A-1090 Vienna, Austria

[4]Eindhoven University of Technology, Department of Applied Physics, Soft Matter and Biological Physics, Postbus 513, NL-5600 MB Eindhoven, The Netherlands

[5]Biomacromolecular Systems and Processes, Institute of Biological Information Processing (IBI-4), 4 Forschungszentrum Jülich, D-52425 Jülich, Germany

This PDF file includes:

Supplementary text
Figures S1 to S5
SI References

Supporting Information Text

Additional exemplary renderings of experimental samples

We report in Fig. S1 exemplary renderings of the experimental sample structures complementing those reported in Fig.3 of the main article. Renderings were reconstructed from the coordinates extracted from particle tracking applied to optical microscopy experiments.

Radial distribution functions $g(r)$ and mean squared displacements for the system with 25mM NaCl

We show in Fig. S2 the $g(r)$ functions obtained from the particle coordinates extracted from microscopy experiments on samples containing 25mM NaCl in solution. Compared to the system without salt, the samples show a gradual and monotonic increase of the order with increasing η, as indicated by the progressive increase of the height of the first peak and the shift of the peak position to increasingly lower values.
The corresponding mean squared displacements are presented in Fig. S3. Similar to the $g(r)$, the MSDs curves for different value of η show a progressive, almost monotonic (except for sample with $\eta = 0.70$) reduction of the displacements.

Derivation of the free energy model

As mentioned in the main text, we include six contributions in the free energy

$$F = U_{\mathrm{H}} + F_{\mathrm{el}} + F_{\mathrm{Fl}} + S_2 + S_3 + F_{\mathrm{p}}.$$

Here, we elaborate on the electrostatic Hartree term U_{H} and the entropic terms S_2 and S_3. Additionally, we provide the analytical expression that results from the minimization procedure with respect to the number of escaped counterions N_3 and the brush height L.

The Hartree energy is given by

$$U_{\mathrm{H}} = \frac{1}{8\pi\varepsilon} \iint \mathrm{d}r\, \mathrm{d}r' \frac{\rho(\mathrm{r})\rho(\mathrm{r}')}{|r - r'|},$$

where

$$\rho(r) = \begin{cases} 0 & r < R_{PS} \\ -\dfrac{eN_3}{4\pi L r^2} & R_{PS} \leq r \leq R \\ \dfrac{3eN_3}{4\pi(R_W^3 - R^3)} & R < r < R_W \end{cases}$$

as explained in the main text. The Hartree energy can now be evaluated directly by substitution of $\rho(r)$. The result is

$$\frac{U_{\mathrm{H}}}{k_B T} = \frac{N_3^2 \lambda_B}{2}\left[\frac{R_{PS}}{L^2}\vartheta_1\left(\frac{R}{R_{PS}}\right) + \frac{1}{R}\vartheta_2\left(\frac{R}{R_W}\right)\right]$$

in which the functions $\vartheta_1(x)$ and $\vartheta_2(x)$ are given by

$$\vartheta_1(x) = x - 2\ln x - \frac{1}{x}, \quad \text{and} \quad \vartheta_2(x) = \frac{5 - 9x + 5x^3 - x^6}{5(1 - x^3)^2}.$$

Since $L = R - R_{PS}$, the derivatives of these functions will appear in the result of the free energy minimization.

The entropic contribution in the free energy can be described as the sum of two contributions. The first, S_2, is the contribution due to the counterions within the brush that are not strongly condensed along the PE-chains. The second, S_3, is the term that corresponds to the counterions that have escaped the brush. Here, we neglect the entropy of the condensed counterions. However, we do take into account that the presence of the PE-chains limits the available free volume to the entropic counterions inside the brush. This can be achieved by introducing a reduced brush thickness L', such that the available volume to the entropically active counterions is reduced by the volume of the PE-chains, which we model as cylindrical.

$$V_2 \equiv \frac{4\pi}{3}(R_{PS} + L')^3 = \frac{4\pi}{4}(R_{PS} + L)^3 - fL\pi R_m^2,$$

where R_{m} is the radius of a chain monomer and V_2 is the volume available to the entropic counterions. The volume available to the counterions outside the brush is simply

$$V_3 = \frac{4\pi}{3}(R_W^3 - R^3).$$

The entropic contributions can now be evaluated from Eq. (9) in the main text

$$\frac{S_2}{k_B T} = N_2\left[1 + \ln\left(\frac{N_2\sigma^3}{4\pi L'(R_{PS} + L')^2}\right) - \frac{2R_{PS}}{L'}\ln\left(\frac{R_{PS} + L'}{R_{PS}}\right)\right]$$

$$\frac{S_3}{k_B T} = N_3\left[\ln\left(\frac{N_3\sigma^3}{V_3}\right) - 1\right].$$

As all contributions to the free energy in our model are now known, we can proceed with the minimization with respect to N_3 and L, under the condition that $N_1 + N_2 + N_3 = Nf$. This ultimately yields the system of equations

$$N_3\lambda_B = \frac{2 + \ln\left(\frac{N_2}{N_3}\frac{R_W^3 - R^3}{3L'(R_{PS}+L')^2}\right) - \frac{2R_{PS}}{L'}\ln\left(\frac{R_{PS}+L'}{R_{PS}}\right) + \frac{R^3}{R_W^3}}{\frac{R_c}{L^2}\vartheta_1\left(\frac{R}{R_{PS}}\right) + \frac{1}{R}\vartheta_2\left(\frac{R}{R_W}\right)},$$

$$\frac{N_3^2\lambda_B}{2} = \frac{\frac{3fL}{Nb^2} - \frac{9vf^2N^2}{8\pi L^4} + \frac{2R_{PS}N_2}{L'^2}\ln\left(\frac{R_{PS}+L'}{R_{PS}}\right) - \frac{3N_2}{L'} + \frac{4\pi R^2 N_3}{V_3} + \frac{3N_2R^2}{R_W^3}}{\frac{R_{PS}}{L^3}\vartheta_1\left(\frac{R}{R_{PS}}\right) - \frac{1}{L^2}\vartheta_1'\left(\frac{R}{R_{PS}}\right) + \frac{1}{R^2}\vartheta_2\left(\frac{R}{R_W}\right) - \frac{1}{RR_W}\vartheta_2'\left(\frac{R}{R_W}\right)},$$

which we solve numerically using Newton's method.

Additional information and results on the molecular dynamics simulations of the miniature brush

To verify the theoretical predictions that we make in the main text, we perform molecular dynamics simulations of miniature brushes. Our simulation model consists of a large colloidal particle to which f charged polymer chains are grafted. The PE-chains are simulated similarly to the model that was used by Wynveen *et al.* (*65*)

We coarse-grain the PE-chains as a single polymer chain consisting of Lennard-Jones monomers that each have an assigned charge $-e$. To ensure charge neutrality, we introduce fN positively charged counterions into the simulation box. All particles interact according to a shifted and truncated Lennard-Jones potential as function of the distance r between the centers of mass of the particles. This potential models the steric interactions of particles in good solvent conditions, and is given by

$$V_{\mathrm{LJ}}^{\mu\nu}(r) = \begin{cases} 4\varepsilon_{LJ}\left[\left(\frac{d}{r-\delta^{\mu\nu}}\right)^{12} - \left(\frac{d}{r-\delta^{\mu\nu}}\right)^{6}\right] + \varepsilon_{LJ} & \text{if} \quad r < \sqrt[6]{2}d + \delta^{\mu\nu} \\ 0 & \text{otherwise} \end{cases}$$

where we set $d = 4.0$ Å, and $\varepsilon_{LJ} = 1.0$ kJ/mol. The indices μ and ν represent the three different species of particles $\mu, \nu \in \{C_0, M_-, m_+\}$, in which C_0 denotes the large central colloid, M_- the chain monomers, and m_+ the free counterions. The shift $\delta^{\mu\nu}$ in the Lennard-Jones potential (not to be confused with a Kronecker delta) is given by $\delta^{\mu\nu} = r^{\mu} + r^{\nu} - d$, where $r^{C_0} = R_{PS} = 100$ Å, $r^{M_-} = R_m = 9.0$ Å, and $r^{m_+} = d/2 = 2.0$ Å. This interaction potential models counterion-counterion interactions with a usual truncated Lennard-Jones potential. All other two-particle interactions are modelled with this same

potential, shifted by $\delta^{\mu\nu}$. This choice implies that the monomer-counterion interaction is equal to ε_{LJ} at $r = 11$ Å, consistent with the steric radius of a DNA-chain.

We model electrostatic interactions between the particles i and j having charge numbers Z_i and Z_j with a Coulomb potential

$$V_C^{ij}(r) = k_B T Z_i Z_j \frac{\lambda_B}{r},$$

in which we treat the solvent implicitly by a dielectric medium at temperature $k_B T = 2.4\varepsilon_{LJ}$ with corresponding Bjerrum length $\lambda_B = 7.1$ Å, characteristic of water at room temperature. The long-range interactions were computed using a three-dimensional Ewald-summation with a relative error smaller than 10^{-4}. Decreasing this value is found to have no significant effect on the simulation results. Periodic boundaries are implemented using the minimum image convention.

The monomers in the chains are connected to their neighbors by harmonic bonds that are described by the harmonic potential

$$V_b(r) = \frac{1}{2} k_H (r - b)^2,$$

where $b = 3.4$ Å and $k_H = 210\,\mathrm{kJ\,mol^{-1}\,Å^{-2}}$. The latter value was chosen such that potential has a strength of $k_B T$ when $|r - b| = 0.15$ Å, which is a measure of the dispersion of the monomers of a chain. Because the equilibrium bond length is much smaller than the distance at which the steric monomer-monomer interaction diverges, we exclude the repulsive interactions of the 8 nearest neighbors on each side of every chain particle. To be able to capture the swelling behavior of the brush due to the osmotic pressure of the counterions and the electrostatic repulsion between the chain monomers, we do not include a valence angle potential.

Using a Langevin thermostat, we effectively perform Brownian dynamics simulations in the canonical ensemble. The time step is set to 10^{-3}, where $\tau = \sqrt{md^2/\varepsilon_{LJ}} \approx 10$ ps, with $m = 660$ g/mol as the mass of a chain monomer, roughly equal to the mass of a DNA base pair. The masses of the colloid and counterions respectively are set to 6.6×10^4 g/mol and 20 g/mol. We set the relaxation time of the Langevin equations to equal τ, which implicitly defines the effective viscosity of the solvent. The simulations were typically run for $\sim 10^6$ time steps, excluding an equilibration time of $10^5 - 10^6$ steps.

In the construction of the theoretical model, a few important assumptions were made. One of those stated that the density of monomers and counterions within the brush decays as r^{-2}, where r is the distance from the brush center. Our simulation results confirm this as is shown in Fig S4. Here we see the monomer and counterion number densities plotted on a double logarithmic scale, and we find that, within the brush, the monomer density decays roughly as $r^{-2.0}$. Note that the exponent of -2 does not strictly mean that the chains are perfectly stretched; a brush with chains that have 'corkscrew' features, or stretched chains

that are not perfectly radially oriented also produce the same exponent in the decay of the monomer density. Both phenomena are to some degree present in the simulation results, as is confirmed by snapshots of the simulation, of which an example is shown in Fig. 2b in the main text.

In Fig. S4, we also observe that the counterion distribution follows the profile of the monomer distribution within the brush to minimize the electrostatic interaction energy. In contradiction to our assumptions, the counterion distribution does not appear to be constant outside of the brush. This has however a negligible effect on the long-range electrostatic interaction and on the entropy S_3. The aforementioned model has been also applied to two interacting miniature brushes to calculate in a molecular dynamics simulation the effective force between them as a function of their separation. Results are shown in Fig. S5 in comparison with the theoretical result, confirming the validity of the latter. Due to its small number of arms, the miniature brush is not osmotic, and thus the interaction is dominated in this case by electrostatics. For the experimental osmotic brushes, on the other hand, the potential is a steeply increasing function of the separation as the latter diminishes beyond brush overlap, as established earlier by Jusufi *et al.* (2)

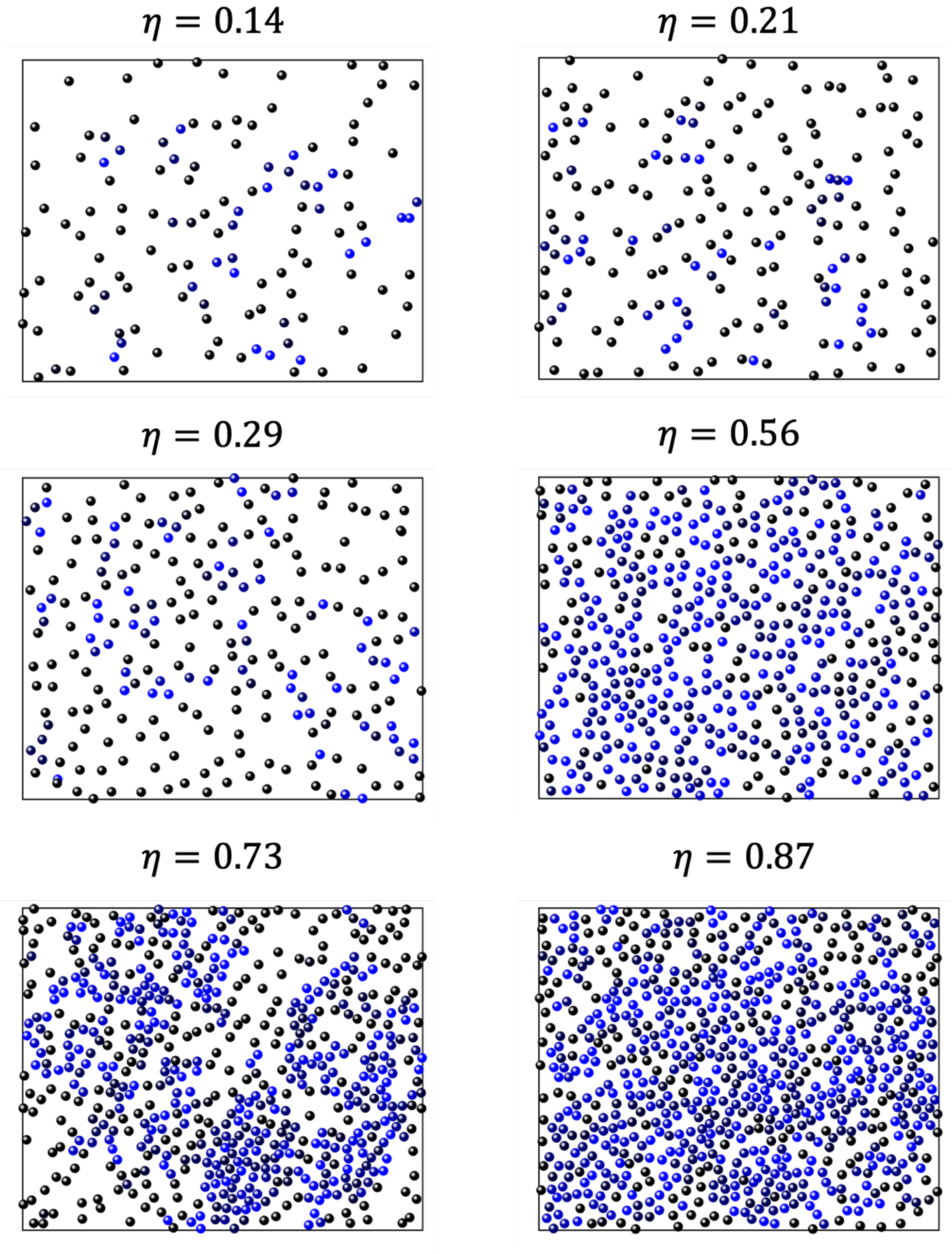

Fig. S1. Renderings of the experimental samples, representative of the structural organization of the dispersion with the corresponding packing fraction $\boldsymbol{\eta}$, as indicated.

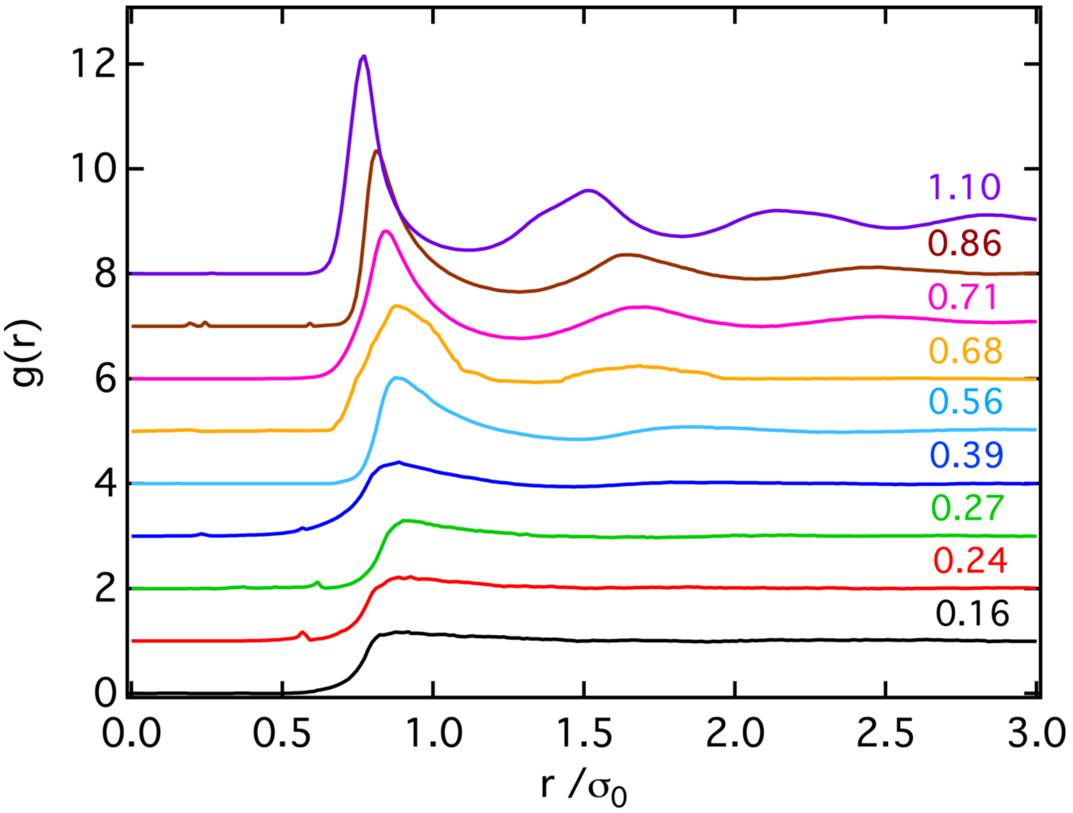

Fig. S2. Radial distribution functions $\boldsymbol{g(r)}$ for the system with 25mM NaCl, for different packing fractions $\boldsymbol{\eta}$, as indicated. The curves are shifted each time by a factor of 1 with respect to the data corresponding to the previous value of $\boldsymbol{\eta}$.

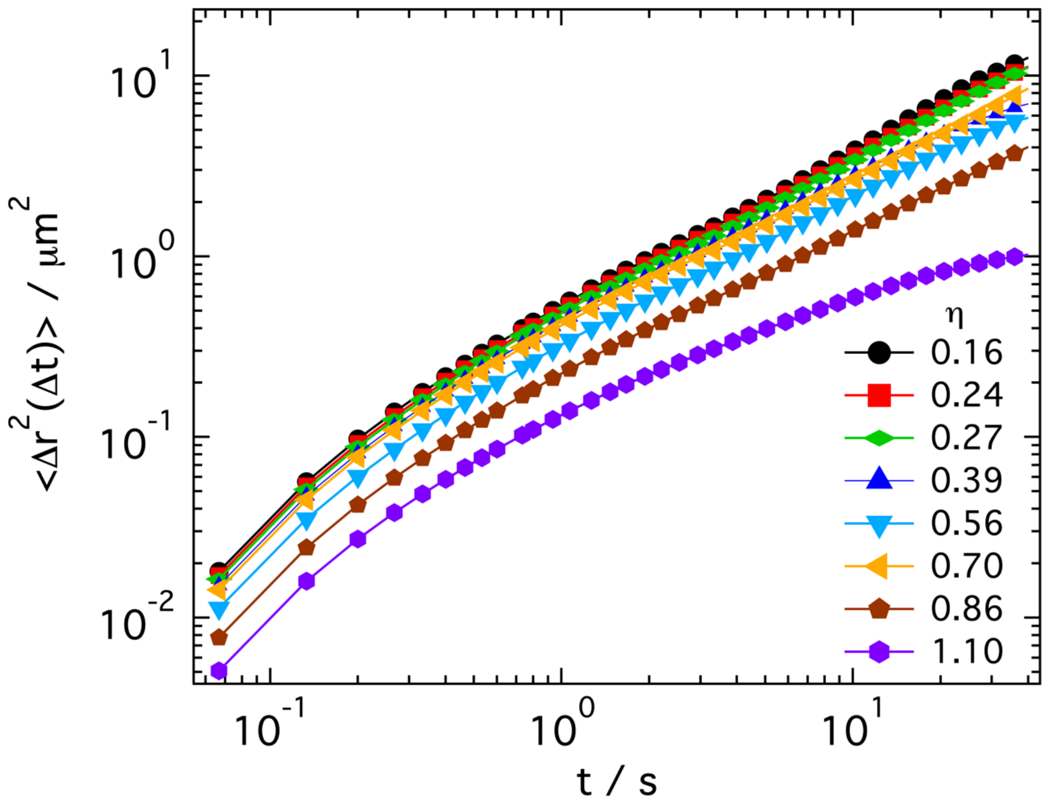

Fig. S3. Mean squared displacements $\langle \Delta r^2(\Delta t) \rangle$ for dispersions of dsDNA colloids with 25mM NaCl in solution. The packing fraction is indicated in the legend.

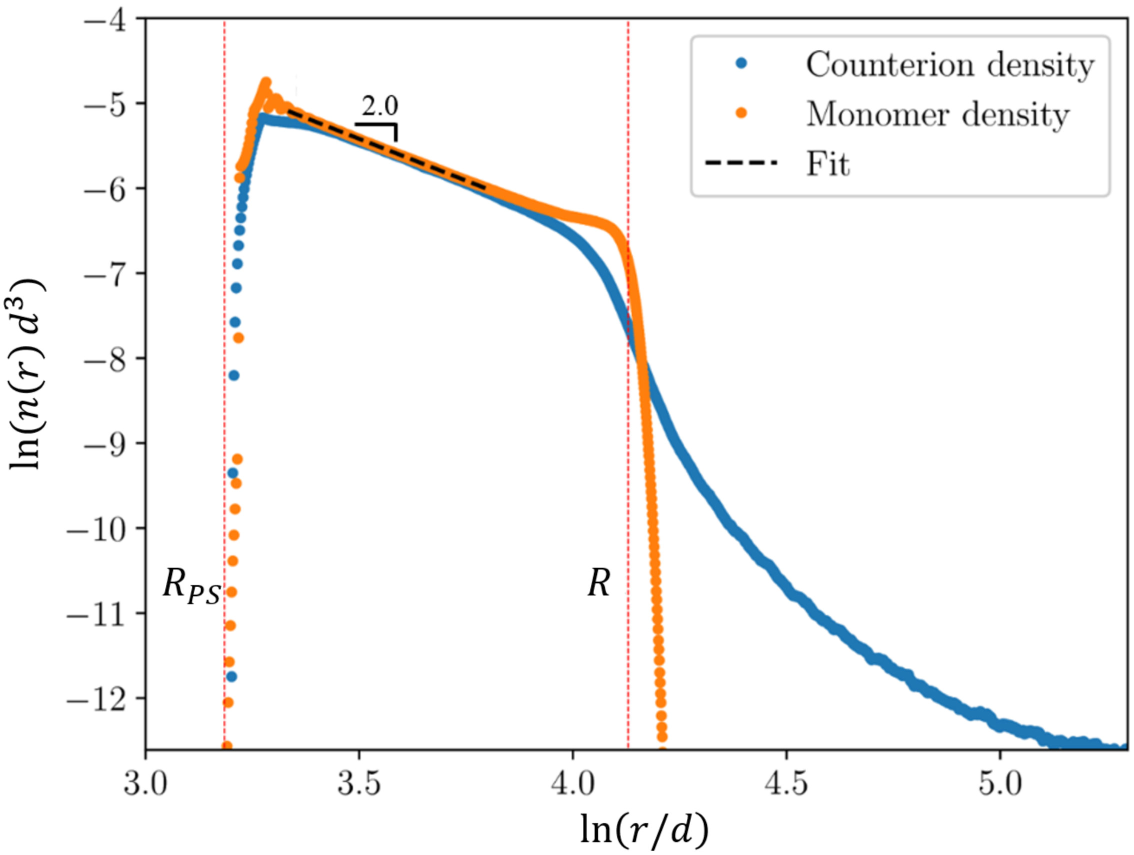

<insert page break here>

Fig. S4. Double logarithmic plot of the monomer and counterion number density as function of the radial distance for a brush with functionality $\boldsymbol{f = 40}$ and chain length $\boldsymbol{N = 60}$ in a cubic box with sides $\boldsymbol{L_x = L_y = L_z = 400}$. The vertical dashed lines indicate the colloid radius $\boldsymbol{R_{PS}}$ and the brush radius $\boldsymbol{R = L + R_{PS}}$, the latter being determined as the average distance the last chain monomers are separated from the brush centre. A straight line was fitted to the monomer density data within the brush. We find a slope of -2.0, indicating the stretched nature of the PE chains.

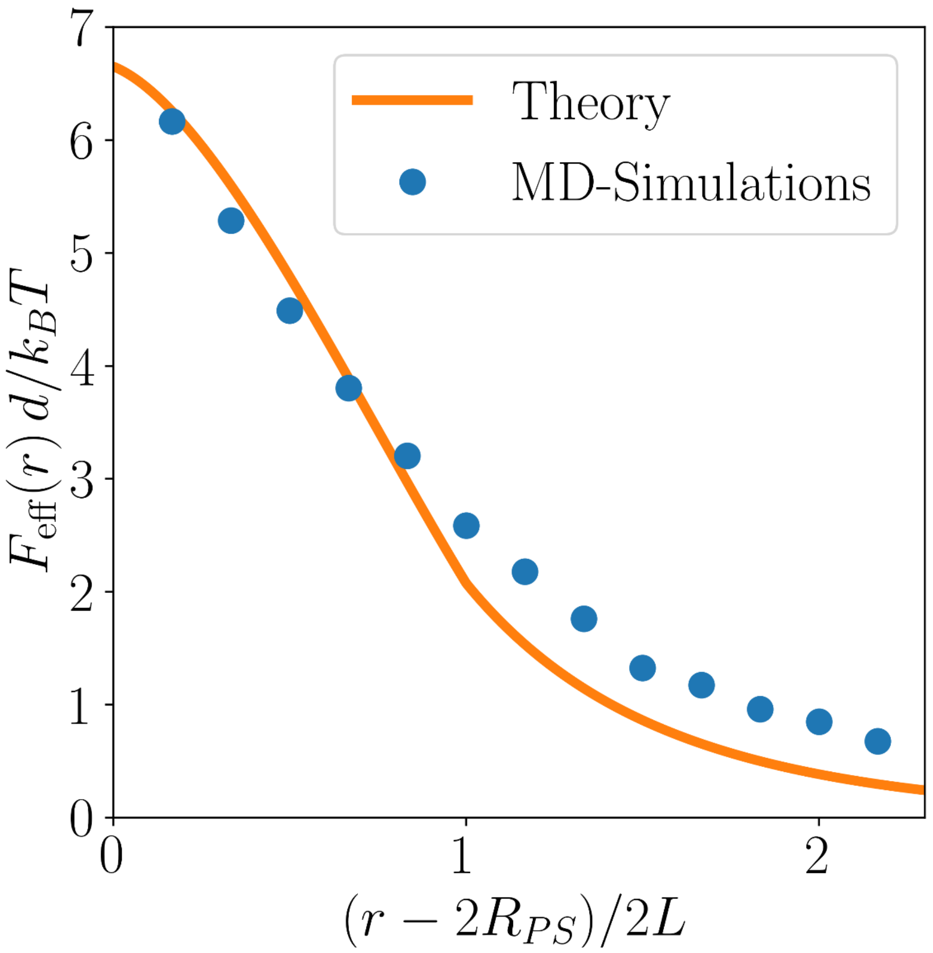

Fig. S5. The effective force $\boldsymbol{F_{\mathrm{eff}}}$ in units of $\boldsymbol{k_BT/d}$ as a function of the separation distance $\boldsymbol{r}$ acting on brushes with functionality $\boldsymbol{f = 20}$ and chain length $\boldsymbol{N = 50}$. We have fixed the box size at $\boldsymbol{L_x/4 = L_y = L_z = 160d}$. The dots are molecular dynamics simulation results, whereas the line represents the predictions from the theory (derivation will be provided upon reasonable request). The horizontal axis is scaled such that a value of 0 indicates that the two colloidal cores are touching, $\boldsymbol{r = 2R_{PS}}$. Similarly, a value of 1 indicates that the brushes are touching but not overlapping, $\boldsymbol{r = 2R}$.

References

1. A. Wynveen, C. N. Likos, Interactions between planar polyelectrolyte brushes: effects of stiffness and salt. *Soft Matter* **6**, 163–171 (2010).
2. A. Jusufi, C. N. Likos, M. Ballauff, Counterion distributions and effective interactions of spherical polyelectrolyte brushes. *Colloid Polym. Sci.* **282**, 910–917 (2004).
